# Harmony Search Method: Theory and Applications

**DOI:** 10.1155/2015/258491

**Published:** 2015-04-07

**Authors:** X. Z. Gao, V. Govindasamy, H. Xu, X. Wang, K. Zenger

**Affiliations:** ^1^Department of Electrical Engineering and Automation, Aalto University School of Electrical Engineering, 00076 Aalto, Finland; ^2^Department of Information Technology, Pondicherry Engineering College, Pondicherry 605 014, India; ^3^College of Mechanical and Electrical Engineering, Harbin Engineering University, Harbin 150001, China

## Abstract

The Harmony Search (HS) method is an emerging metaheuristic optimization algorithm, which has been employed to cope with numerous challenging tasks during the past decade. In this paper, the essential theory and applications of the HS algorithm are first described and reviewed. Several typical variants of the original HS are next briefly explained. As an example of case study, a modified HS method inspired by the idea of Pareto-dominance-based ranking is also presented. It is further applied to handle a practical wind generator optimal design problem.

## 1. Introduction

Firstly proposed by Geem et al. in 2001 [1], the Harmony Search (HS) method is inspired by the underlying principles of the musicians' improvisation of the harmony. The HS has the distinguishing features of algorithm simplicity and search efficiency. During the recent years, it has been successfully used in areas such as function optimization [2], mechanical structure design [3], pipe network optimization [4], and optimization of data classification systems [5]. In this paper, we first introduce the underlying inspiration and principles of the basic HS method in Section 2. Some representative modified HS algorithms are next explained in Section 3. The applications of the HS in various real-world areas are surveyed and reviewed in the following section. In Section 4, to demonstrate an illustrative example, we present and discuss a new HS method for the constrained optimization with application in the wind generator design. Finally, in Section 5, the paper is concluded with some remarks and conclusions.

## 2. Harmony Search Method

**Figure 1:**
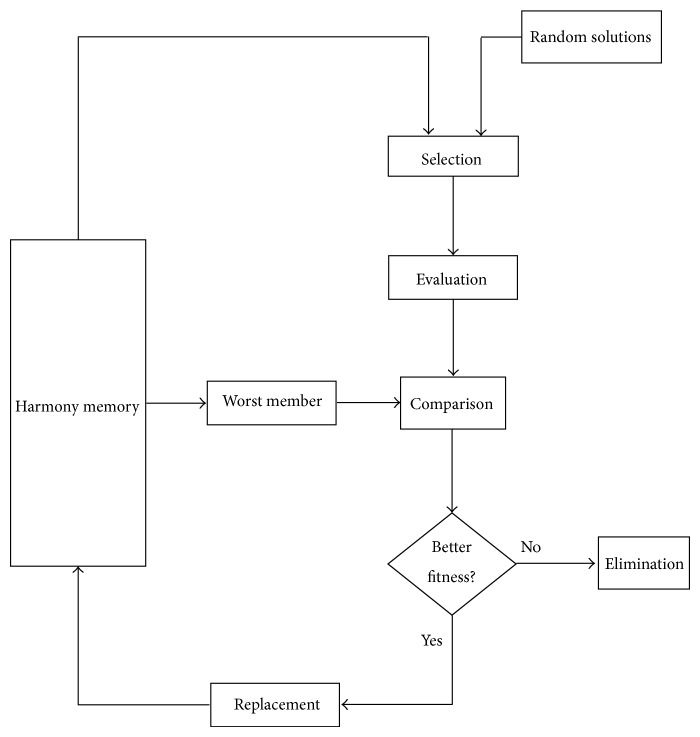
Harmony Search (HS) method.

As we know, when musicians compose the harmony, they usually try various possible combinations of the music pitches stored in their memory. This search for the perfect harmony is indeed analogous to the procedure of finding the optimal solutions to engineering problems. The HS method is actually inspired by the working principles of the harmony improvisation [1].  Figure 1 shows the flowchart of the basic HS method, in which there are four principal steps involved. The pseudocode of the HS is given in Algorithm 1.

**Step 1 .**

Initialize the HS Memory (HM). The initial HM consists of a certain number of randomly generated solutions to the optimization problems under consideration. For an *n*-dimension problem, an HM with the size of *N* can be represented as follows:

(1) $$\begin{array}{c} \text{HM}=\left[\begin{array}{c} x_{1}^{1},x_{2}^{1},\ldots ,x_{n}^{1}\\ x_{1}^{2},x_{2}^{2},\ldots ,x_{n}^{2}\\ \vdots \\ x_{1}^{\text{HMS}},x_{2}^{\text{HMS}},\ldots ,x_{n}^{\text{HMS}} \end{array}\right], \end{array}$$

where [*x*_1_^*i*^, *x*_2_^*i*^,…, *x*_*n*_^*i*^] (*i* = 1,2,…, HMS) is a solution candidate. HMS is typically set to be between 50 and 100.

**Step 2 .**

Improvise a new solution [*x*_1_′, *x*_2_′,…, *x*_*n*_′] from the HM. Each component of this solution, *x*_*j*_′, is obtained based on the Harmony Memory Considering Rate (HMCR). The HMCR is defined as the probability of selecting a component from the HM members, and 1-HMCR is, therefore, the probability of generating it randomly. If *x*_*j*_′ comes from the HM, it is chosen from the *j*th dimension of a random HM member and is further mutated according to the Pitching Adjust Rate (PAR). The PAR determines the probability of a candidate from the HM to be mutated. As we can see, the improvisation of [*x*_1_′, *x*_2_′,…, *x*_*n*_′] is rather similar to the production of offspring in the Genetic Algorithms (GAs) [6] with the mutation and crossover operations. However, the GA creates new chromosomes using only one (mutation) or two (simple crossover) existing ones, while the generation of new solutions in the HS method makes full use of all the HM members.

**Step 3 .**

Update the HM. The new solution from Step 2 is evaluated. If it yields a better fitness than that of the worst member in the HM, it will replace that one. Otherwise, it is eliminated.

**Step 4 .**

Repeat Step 2 to Step 3 until a preset termination criterion, for example, the maximal number of iterations, is met.

Similar to the GA and swarm intelligence algorithms [7], the HS method is a random search technique. It does not require any prior domain knowledge, such as the gradient information of the objective functions. However, different from those population-based approaches, it only utilizes a single search memory to evolve. Therefore, the HS method has the distinguishing feature of computational simplicity.

## 3. Variants of HS Method

A lot of modified HS algorithms have been studied in the past decade so as to enhance the performances of the original version. As a matter of fact, a special discrete variation of the HS is proposed by Geem on the basis of introducing the stochastic derivatives for the discrete variables involved [8]. The stochastic derivatives give the selection probabilities of certain discrete variables during the evolution procedure of the HS. It is efficient at manipulating discrete optimization problems and has been employed in the optimal design of fluid-transport networks. Omran and Mahdavi embed the ideas borrowed from swarm intelligence into the regular HS and develop a new variant: global-best HS (GHS) [9]. In the GHS, the adjustment of new solutions improvised is only based on the best harmony selected from the HM without the involvement of the distance bandwidth (bw). This interesting approach adds the unique social learning capability to the GHS. The investigation experiments of ten benchmark functions prove that the GHS can generally outperform the original HS. Inspired by the local versions of the Particle-Swarm Optimization (PSO) [10] and GHS, Pan et al. propose a local-best variant of the HS method with dynamic subpopulation: DLHS [11]. In the DLHS, the whole HM is divided into multiple sub-HMs, each of which can evolve independently. However, these sub-HMs will form the HM again after searching for the optimal solutions in their own regions. With this subpopulation policy and a simple local search strategy, the DLHS is capable of achieving a satisfactory compromise between the exploration and exploitation in search. It has been successfully applied to attack the difficult lot-streaming flow shop scheduling problem. Inspired by the GHS and DLHS, Geem further develops the Particle-Swarm Harmony Search (PSHS) [12], which has been validated to be better than the original HS algorithm for small-size problems but worse in case of large-scale one. A few novel HS methods have also been introduced by the authors of the present paper [13–17].

The parameters of HMCR and PAR usually play a critical role in the optimization performance of the HS method. Unfortunately, properly choosing the appropriate values for them is always a challenging topic. Mahdavi et al. study an adaptive strategy for adjusting PAR and bw in the Improved HS (IHS) algorithm [18]. The values of PAR and bw dynamically increase and decrease with the growth of HS iterations, respectively, so as to enhance the performance of the IHS. The IHS has been demonstrated to achieve comparable performances with other evolutionary and mathematical programming techniques in dealing with several test problems in terms of both the number of the fitness function evaluations required and the quality of the solutions found. Unfortunately, the lower and upper limits for the update of PAR and bw are often case dependent and are therefore difficult to determine. In [19], another adaptive HS method is proposed and explored. It takes advantage of two varying control parameters, *η* and *ρ*, to generate new harmony vectors. Both of these parameters are selected from the average values that are observed within the current harmony memory matrix using a given probability density function. This adaptive HS algorithm has found great successes in handling large steel structure optimization problems. Wang and Huang propose a new self-adaptive HS technique in [20]. Their almost parameter-free HS uses the information stored in the HM (self-consciousness), that is, the minimum and maximum of the present HM members, to automatically control the pitch adjustment step. The low-discrepancy sequences are also utilized to initialize the HM. It has been compared with the aforementioned IHS and GHS and can offer superior performances on four optimization problems tested. Geem and Sim introduce the Parameter-Setting-Free (PSF) technique to eliminate the common difficulty of selecting suitable HS parameters [21]. The developed PSF-HS has a new operation step, namely, rehearsal, in which certain numbers of new solutions are generated with the initial HMCR and PAR. The adaptive HMCR and PAR are then calculated based on the rehearsal results evaluated. The PSF-HS has been shown to be more robust than the original HS method, although its computational complexity is moderately high. The authors of the present paper also study a fusion of the HS and Cultural Algorithm (CA), HS-CA, in which the search knowledge stored in the CA is utilized to guide the mutation direction and size of the HS. This HS-CA is further used to effectively cope with an optimal wind generator design problem [15]. In [16], a hybrid HS method inspired by the opposition-based learning is proposed by the same authors. The HS method is merged with the Population-Based Incremental Learning (PBIL) for the optimal design of electrical machines in [17].

Theoretical research on the working principles and search mechanism of the HS method has been reported in the recent literature, which can provide a useful guideline for users to design this algorithm in practice. Das et al. discuss the exploratory power of the HS by analyzing the evolution of the population variance over successive generations of the HM [22]. Based on their analysis work, they further propose a modified HS algorithm, Exploratory HS (EHS), in which bw for the pitch adjustment is set to be proportional to the standard deviation of the HM population. In the simulation study, the EHS can not only outperform three existing HS variants, IHS, GHS, and MHS [23], over all the test functions, but also yield better or at least comparable results when compared with a few state-of-the-art swarm intelligence techniques. Unfortunately, how to choose the optimal proportional gain *k* for bw in the EHS is still an open issue.

## 4. Applications of HS Method

In the real world, modern science and industry are indeed rich in the problems of optimization. Since the HS has been originally proposed by Geem and applied to solve the optimization problem of water distribution networks in 2000, the applications of the HS have covered numerous areas including industry, optimization benchmarks, power systems, medical science, control systems, construction design, and information technology [24].

### 4.1. Optimization Benchmarks

Optimization benchmarks for the hybridization of the HS method with other approaches are one principal application area. Different variants based on the HS have demonstrated their improvement and efficiency through various benchmark functions. Combined with semantic genetic operators, a Geometric Selective Harmony Search (GSHS) method was proposed by Castelli et al. with three main differences from the original HS: (1) the memory consideration process involves the presence of a selection procedure, (2) the algorithm integrates a particular recombination operator that combines the information of two harmonies, and (3) the algorithm utilizes a mutation operation that uses the PAR parameter. Therefore, geometric semantic crossover produces offspring that is not worse than the worst of its parents, and geometric semantic mutation causes a perturbation on the semantics of solutions, whose magnitude is controlled by a parameter. Five different HS algorithms have been compared using 20 benchmark problems, and the GSHS outperforms the others with statistically significant enhancement in almost all the cases [25].

### 4.2. Industry

Industry is a prominent area full of various multimodal, constrained, nonlinear, and dynamical optimization problems. The HS algorithm proposed by Saka [26] determines the optimal steel section designations from the available British steel section table and implements the design constraints from BS5950. Recently, an Enhanced Harmony Search (EHS) in [27] is developed enabling the HS algorithm to quickly escape from local optima. The proposed EHS algorithm is utilized to solve four classical weight minimization problems of steel frames including two-bay, three-storey planar frame subject to a single-load case, one-bay, ten-storey planar frame consisting of 30 members, three-bay, twenty-four-storey planar frame, and spatial 744-member steel frame. In [28], the HS is used to select the optimal parameters in the tuned mass dampers. Fesanghary et al. propose a hybrid optimization method based on the global sensitivity analysis and HS for the optimal design of shell and tube heat exchangers [29].

### 4.3. Power Systems

There is a lot of work focused on the optimization issues concerning power systems, such as cost minimization. A modified HS algorithm is proposed to handle nonconvex economic load dispatch of real-world power systems. The economic load dispatch and combined economic and emission load dispatch problems can be converted into the minimization of the cost function [30]. Sinsuphan et al. combine the HS with sequential quadratic programming and GA to solve the optimal power flow problems. The objective function to be optimized is the total generator fuel costs in the entire system [31]. The chaotic self-adaptive differential HS algorithm, proposed by Arul et al., is employed to deal with the dynamic economic dispatch problem [32].

### 4.4. Signal and Image Processing

Li and Duan modify the HS by adding a Gaussian factor to adjust the bw. With this modified HS, they develop a pretraining process to select the weights used in the combining of feature maps to make the target more conspicuous in the saliency map [33]. In their method based on the HS, Fourie et al. design a harmony filter using the Improved HS algorithm for a robust visual tracking system [34].

### 4.5. Others

In addition to the aforementioned applications, the HS has also been widely employed in a large variety of fields, including transportation, manufacturing, robotics, control, and medical science [24]. Xu et al. explore the applications of the HS in the prototype optimization and selection of the reconfigurable mobile robots in the sandy terrain [35, 36]. Many traffic modeling software packages are capable of finding the optimal or near-optimal signal timings using different optimization algorithms. For example, Ceylan proposes a modified HS with embedded hill climbing algorithm for further tuning the solutions in the stochastic equilibrium network design [37]. The modified HS algorithm is also used in parameter identification of the solar cell mathematical models [38]. Miguel et al. employ the HS method in damage detection under the ambient vibration [39].

## 5. A Modified HS Method for Constrained Optimization: A Case Study

### 5.1. Constrained Optimization Problems

Most of the practical optimization problems are indeed constrained optimization problems, whose goal is to find the optimal solution that satisfies a set of given constraints. In general, a constrained optimization problem is described as follows.

Find $\vec{x}=(x_{1},x_{2},\ldots ,x_{n})$ to minimize $f(\vec{x})$, subject to $g_{i}(\vec{x})\leq 0$, *i* = 1,2,…, *M*, and $h_{j}(\vec{x})=0$, *j* = 1,2,…, *N*, where $f(\vec{x})$ is the objective function and $g_{i}(\vec{x})$ and $h_{j}(\vec{x})$ are the inequality and equality constraint functions, respectively. As a matter of fact, the equality constraint functions can be transformed into the inequality constraint functions:

(2) $$\begin{array}{c} \left|h_{j}\left(\vec{x}\right)\right|-\varepsilon \leq 0, \end{array}$$

where *ε* is a small enough tolerance parameter. Therefore, we only consider the inequality constraint functions $g_{i}(\vec{x})\leq 0$, *i* = 1,2,…, *M*, here.

The constrained optimization problems are generally difficult to deal with, because the constraint functions can divide the whole search space into some disjoint islands. Numerous constraint-handling techniques have been proposed and investigated during the past decades [40–44]. One popular solution is to define a new fitness function $F(\vec{x})$ to be optimized [41]. $F(\vec{x})$ is the combination of the objective function $f(\vec{x})$ and weighted penalty terms $P_{i}(\vec{x})$, *i* = 1,2,…, *M*, which reflect the violation of the constraint functions:

(3) $$\begin{array}{c} F\left(\vec{x}\right)=f\left(\vec{x}\right)+\overset{M}{\underset{i=1}{\sum }}w_{i}P_{i}\left(\vec{x}\right), \end{array}$$

where *w*_*i*_ (*i* = 1,2,…, *M*) are the preset weights. The overall optimization performance depends on the penalty terms and their weights and may significantly deteriorate with inappropriately chosen ones. Unfortunately, there is no analytic way yet to find the best $P_{i}(\vec{x})$ and *w*_*i*_. In this section, on the basis of the Pareto-dominance, we present a modified HS method for the direct handling of these constraints.

### 5.2. A Modified HS Method for Constrained Optimization

**Figure 2:**
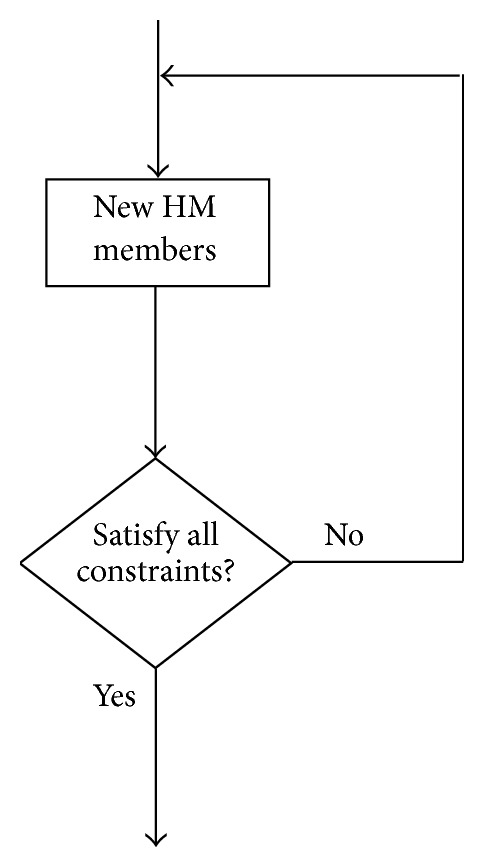
Generation of new HM members satisfying constraints in HS method.

It is well known that the regular HS method is not efficient in attacking the constrained optimization problems. As aforementioned, the HM only stores the feasible solution candidates. The new HM members are generated either from the existing HM members or in a random way. Nevertheless, they are not guaranteed to always meet all the constraints.  Figure 2 shows that, in the HS method, the new HM members satisfying the constraints can be obtained based on only* trial and error*, which may lead to a time consuming procedure, especially in case of complex constraint functions.

**Figure 3:**
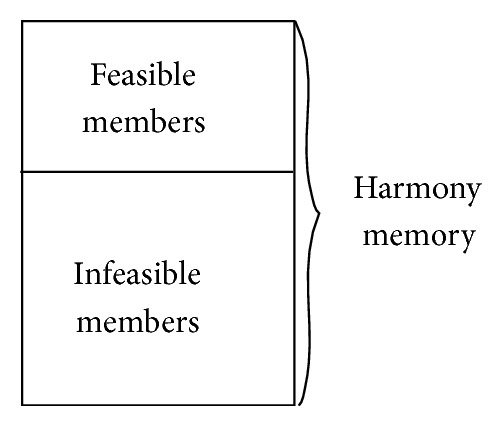
Harmony memory with feasible and infeasible members.

In our modified HS method [14], we make full use of those HM members that do not even meet the constraints. The key issue is how to rank the HM members according to their objective as well as constraint function values. Here, the values of the constraint functions of all the HM members are stored together with their objective function values in the HM. The HM members are divided into two different parts: feasible members and infeasible members, as illustrated in Figure 3. The former satisfy all the constraint functions, while the latter do not. Thus, the ranking of the HM members is separated into two consecutive stages: ranking of the feasible HM members and ranking of the infeasible ones. The ranking of the feasible HM members is straightforward: they can be sorted using their objective function values. However, for the infeasible ones, the ranking is based on the Pareto-dominance of these HM members [42]. An infeasible HM member dominates another, if none of its constraint function values is larger and at least one is smaller. Formally, the Pareto-dominance is defined as follows. Suppose there are two infeasible HM members, ${\vec{x}}^{1}$ and ${\vec{x}}^{2}$. If

(4) ∀i∈1,2,…,M:gix→1≤gix→2∧∃i∈1,2,…,M:gix→1<gix→2,

we conclude that ${\vec{x}}^{1}$ dominates ${\vec{x}}^{2}$. For each infeasible HM member, we can calculate the number of the others that dominate it, which implies its relative degree of violation of the constraint functions. That is, the rank of an infeasible HM member is determined by the number of other infeasible HM members by which it is dominated.

After the HM has been ranked, the worst HM member ${\vec{x}}^{\#}$ can be selected and compared with the new solution candidate ${\vec{x}}^{*}$. Note that ${\vec{x}}^{*}$ does not need to be feasible. When ${\vec{x}}^{\#}$ is compared with ${\vec{x}}^{*}$, ${\vec{x}}^{*}$ will replace ${\vec{x}}^{\#}$ only in one of the following three cases:

1. ${\vec{x}}^{*}$ is feasible, and ${\vec{x}}^{\#}$ is infeasible,
2. both ${\vec{x}}^{*}$ and ${\vec{x}}^{\#}$ are feasible, and $f({\vec{x}}^{*})<f({\vec{x}}^{\#})$,
3. both ${\vec{x}}^{*}$ and ${\vec{x}}^{\#}$ are infeasible, and ${\vec{x}}^{*}$ dominates ${\vec{x}}^{\#}$.

More precisely, ${\vec{x}}^{*}$ replaces ${\vec{x}}^{\#}$ on the condition that

(5) ∀i∈1,2,…,M:gi(x→∗)≤0 ∧∃i∈1,2,…,M:gi(x→#)>0 ∨∀i∈1,2,…,M:gi(x→∗)≤0 ∧∀i∈1,2,…,M:gi(x→#)≤0 ∧fx→∗<fx→# ∨∃i∈1,2,…,M:gi(x→∗)>0 ∧∃i∈1,2,…,M:gi(x→#)>0 ∧∀i∈1,2,…,M:gi(x→∗)≤gi(x→#) ∧∃i∈1,2,…,M:gi(x→∗)<gi(x→#).

**Figure 4:**
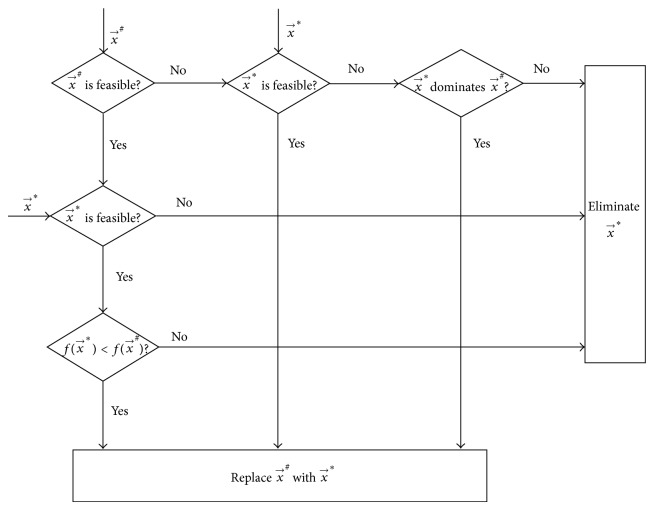
Comparison between ${\vec{x}}^{\#}$ and ${\vec{x}}^{*}$, replacement of ${\vec{x}}^{\#}$ with ${\vec{x}}^{*}$, and elimination of ${\vec{x}}^{*}$.

Figure 4 illustrates this procedure of comparison between ${\vec{x}}^{\#}$ and ${\vec{x}}^{*}$, replacement of ${\vec{x}}^{\#}$ with ${\vec{x}}^{*}$, and elimination of ${\vec{x}}^{*}$.

It is observed from the above descriptions that the infeasible HM members violating the given constraints can also evolve in the modified HS method. In other words, we do not have to always search for new feasible HM members by repeatedly examining them with the constraint functions, as in Figure 2. Compared with the original HS method, our approach needs only a considerably smaller number of constraint function evaluations and, thus, has a lower computational complexity.

### 5.3. Wind Generator Design

**Figure 5:**
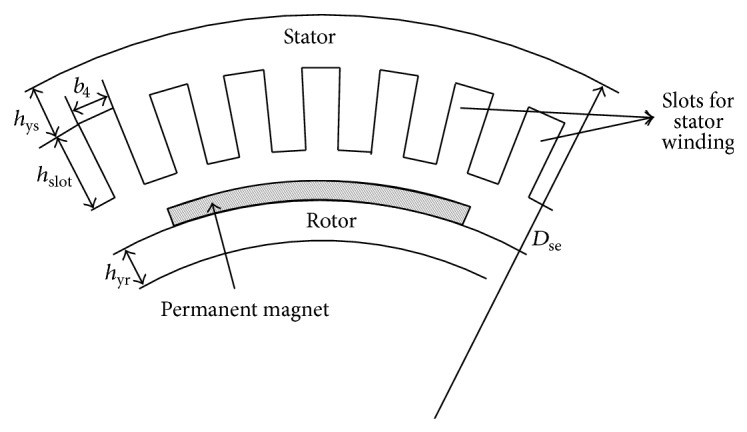
Cross section and dimensions of permanent magnet generator.

**Figure 6:**
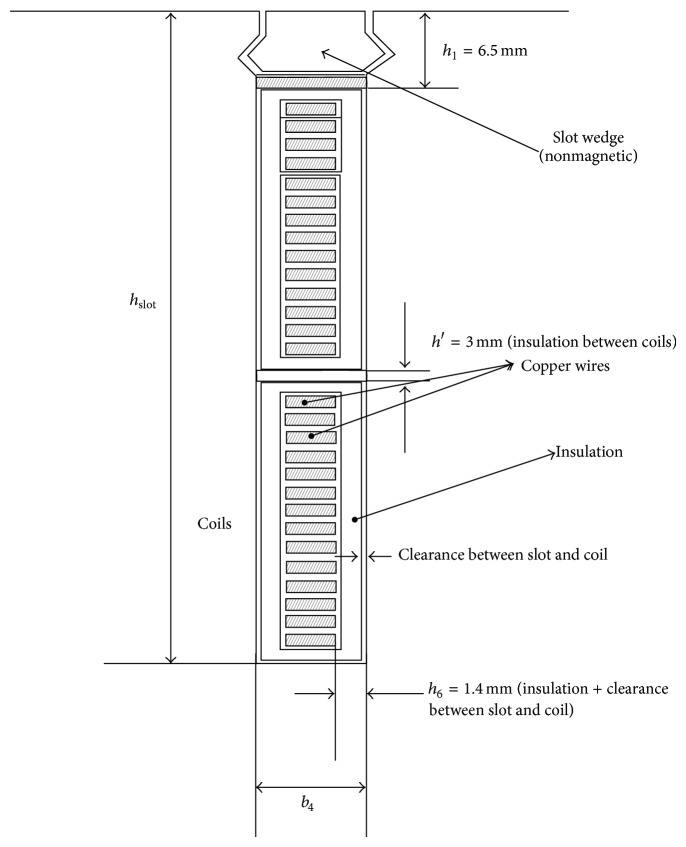
Slot form and constant dimensions of slot.

In this section, we investigate the constrained optimization effectiveness of the modified HS method using a real-world design problem. Wind generator design has been an important but difficult topic in the modern electrical machinery industry. The wind generator shown in Figure 5 is a radial flux type permanent magnet generator, in which the NdFeB magnets are surface-mounted [45]. The remanence flux density of the magnets is 1.05 T, and coercivity 800 kA/m. The stator winding is a three-phase two-layer full-pitch diamond winding. The number of the slots per pole and phase is 2. The stator slot and constant dimensions of the slot are illustrated in Figure 6. The iron core consists of 55 mm long subcores, between which there are radial 6 mm wide ventilation ducts. The length of the subcore is constant, and the number of the ventilation ducts is a decimal fraction in the calculations. The stator frame, bearing shields, and rotor steel body are all 20 mm thick. In the rotor body disc, there are holes, and around 50% of the disc is iron and 50% holes. The iron loss factor is *p*_15_ = 6.6 W/kg with 50 Hz and 1.5 T, and the air-gap length is 5 mm. The rated values of this practical wind generator are given in Table 1.

The detailed design principles of the above wind generator are explained in [45]. The objective function *f*(**x**) (in €) to be minimized is the sum of the material costs and capitalized costs of the total losses of this generator:

(6) fx=kFemFe+kCumCu+kPMmPM+kFefmFrame+kLossPtot,

where *m*_Fe_, *m*_Cu_, *m*_PM_, and *m*_Frame_ are the masses of the stator iron core, stator winding, permanent magnets, and stator frame and rotor body, respectively, *k*_Fe_, *k*_Cu_, *k*_PM_, and *k*_Fef_ the unit prices of the stator core, copper, permanent magnets, and stator frame and rotor body, respectively, and *k*_Loss_ capitalized costs of the losses (Table 2). More details of the calculation of (6) can be found in [45]. The cost of the stator core actually includes the punching, waste parts of the sheet, and assembly of the stator core. The manufacturing cost of the winding is taken into account in the copper cost. The permanent magnet cost includes the corrosion protection, assembly into bigger cassettes, and magnetization of the magnets. The stator frame and rotor body costs consist of the material cost and cost of manufacturing the frame and body.

The stator resistive losses are calculated at the temperature of 100°C, and the iron losses in the stator teeth are

(7) $$\begin{array}{c} P_{\text{Fed}}=2\cdot p_{15}{\left(\frac{B_{d}}{1.5\;\text{T}}\right)}^{2}{\left(\frac{f}{50\;\text{Hz}}\right)}^{1.5}m_{d}, \end{array}$$

where *p*_15_ is the iron loss factor, *B*_*d*_ the maximum flux density in the teeth, *f* the frequency, and *m*_*d*_ the mass of the stator teeth. The iron losses in the stator yoke are

(8) $$\begin{array}{c} P_{\text{Fey}}=1.5\cdot p_{15}{\left(\frac{B_{y}}{1.5\;\text{T}}\right)}^{2}{\left(\frac{f}{50\;\text{Hz}}\right)}^{1.5}m_{y}, \end{array}$$

where *B*_*y*_ is the maximum flux density in the yoke and *m*_*y*_ the mass of the yoke. The losses in the permanent magnets are assumed to be 1% of the rated power, that is, 30 kW. The additional losses are assumed to be 3% of the rated power, that is, 90 kW. The friction and ventilation losses are

(9) $$\begin{array}{c} P_{\rho }=10\cdot D_{r}\left(l+0.6\cdot \tau_{p}\right){\left(\pi nD_{r}\right)}^{2}\left[\text{W}\right], \end{array}$$

where *D*_*r*_ is the outer rotor diameter, *τ*_*p*_ the pole pitch, and *n* the rotational speed of the rotor.  Table 3 gives the nine design parameters to be optimized and their valid ranges.

Like most of the practical design problems, the design variables of this wind generator are also subject to constraints. A total of five given constraints are provided in Table 4. It can be observed that among them there are one constraint on the stator tooth width, three constraints on the flux density of stator and rotor yoke and stator tooth, and one constraint on the output power of the wind generator. Therefore, these constraints must be satisfied by the optimized design variables.

### 5.4. Modified HS Method-Based Optimal Design of Wind Generator

Both the original and our modified HS methods are applied to attack the above demanding wind generator optimal design problem with constraints. The optimization coefficients used are chosen as follows: HMCR = 0.8 and PAR = 0.75. To assess the constrained optimization capabilities of these two HS methods, the Number of Constraint Evaluations (NCEs) is used as the performance criterion here. In other words, the smaller the NCEs are, the more efficient the optimization method is. The evolution procedures of the two HS methods are terminated, when a preset optimization design goal is met, and the NCEs used are compared with each other. In our simulations, there are six optimization goals, that is, 6.65 × 10^5^, 6.625 × 10^5^, 6.6 × 10^5^, 6.575 × 10^5^, 6.55 × 10^5^, and 6.625 × 10^5^. After a total of 1,000 independent trials have been run, the average NCEs used by the original and modified HS methods are given in Table 5. Apparently, the modified HS method uses less NCEs than the original HS for reaching the same optimal design goals. That is to say, the former is more efficient than the latter in coping with the given constraints in this optimal wind generator design problem. It is also worth noting that the improvement of the NCEs usage grows with the increase of the optimization goal.

## 6. Conclusions

In this paper, we provide an overview of the theory and applications of the HS method. The fundamentals of the HS are first introduced. Next, both the basic HS algorithm and some typical variants are discussed in detail. The applications of the HS method in a few applications areas, such as optimization, power systems, signal processing, and robotics, are also presented. Furthermore, as a case study example, a modified HS proposed by the authors for coping with the constrained optimization problems is demonstrated. Based on the Pareto-dominance ranking of the HM members, the given constraints can be directly handled in this new HS method. A real-world optimal wind generator design problem has been employed to verify its effectiveness.

## Figures and Tables

**Algorithm 1:**
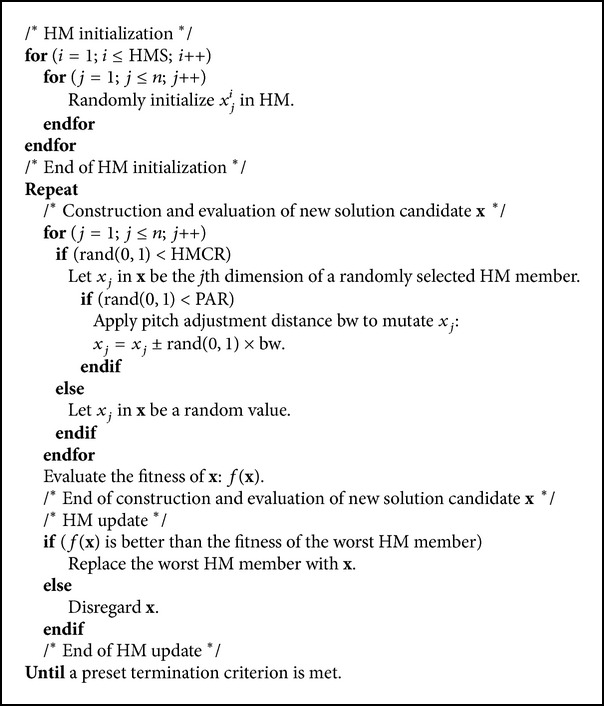
Pseudocode of HS method.

**Table 1:** Rate values of wind generator.

Power	3 MW
Voltage	690 V
Connection	Star
Speed	16.98 rpm
Number of phases	3

**Table 2:** Unit prices of materials and capitalized loss costs.

Electrical steel, *k*_Fe_	4€/kg
Copper, *k*_Cu_	12€/kg
NdFeB magnets, *k*_PM_	60€/kg
Stator frame and rotor steel body, *k*_Fef_	2€/kg
Losses, *k*_Loss_	2€/W

**Table 3:** Wind generator design parameters with ranges.

Parameters	Symbols	Ranges
Stator core length including ventilation ducts	*l*	0.3–3.0 m
Stator yoke height	*h*_ys_	0.01–0.5 m
Stator outer diameter	*D*_se_	3.0–8.0 m
Stator slot height	*h*_slot_	0.07–0.3 m
Maximum flux density in air gap	*B*_max⁡_	0.4–0.9 T
Number of effective conductors in stator slot	*z*_s_	8–26
Rotor yoke height	*h*_yr_	0.01–0.5 m
Number of poles pairs	*p*	20–80
Stator slot width	*b*_4_	0.007–0.04 m

**Table 4:** Wind generator optimization constraints.

Stator tooth width	>8 mm
Stator yoke flux density	<2.2 T
Rotor yoke flux density	<2.2 T
Stator tooth flux density	<2.2 T
Maximum output power	>4.8 MW

**Table 5:** NCEs used by two HS methods for achieving optimization goals.

Optimization goals	HS	Modified HS	Improvement percentages
6.65 × 10^5^	1.8874 × 10^4^	1.2193 × 10^4^	35.40%
6.625 × 10^5^	2.4925 × 10^4^	1.5694 × 10^4^	37.04%
6.6 × 10^5^	3.2848 × 10^4^	1.8874 × 10^4^	42.54%
6.575 × 10^5^	4.2013 × 10^4^	2.3214 × 10^4^	44.75%
6.55 × 10^5^	6.0106 × 10^4^	2.9916 × 10^4^	50.23%
6.625 × 10^5^	8.8975 × 10^4^	4.0342 × 10^4^	54.66%
